# Pressure-induced superconductivity at 32 K in MoB_2_

**DOI:** 10.1093/nsr/nwad034

**Published:** 2023-02-14

**Authors:** Cuiying Pei, Jianfeng Zhang, Qi Wang, Yi Zhao, Lingling Gao, Chunsheng Gong, Shangjie Tian, Ruitao Luo, Mingtao Li, Wenge Yang, Zhong-Yi Lu, Hechang Lei, Kai Liu, Yanpeng Qi

**Affiliations:** School of Physical Science and Technology, ShanghaiTech University, Shanghai 201210, China; Department of Physics and Beijing Key Laboratory of Opto-electronic Functional Materials & Micro-nano Devices, Renmin University of China, Beijing 100872, China; School of Physical Science and Technology, ShanghaiTech University, Shanghai 201210, China; Department of Physics and Beijing Key Laboratory of Opto-electronic Functional Materials & Micro-nano Devices, Renmin University of China, Beijing 100872, China; ShanghaiTech Laboratory for Topological Physics, ShanghaiTech University, Shanghai 201210, China; School of Physical Science and Technology, ShanghaiTech University, Shanghai 201210, China; School of Physical Science and Technology, ShanghaiTech University, Shanghai 201210, China; Department of Physics and Beijing Key Laboratory of Opto-electronic Functional Materials & Micro-nano Devices, Renmin University of China, Beijing 100872, China; Department of Physics and Beijing Key Laboratory of Opto-electronic Functional Materials & Micro-nano Devices, Renmin University of China, Beijing 100872, China; Department of Physics and Beijing Key Laboratory of Opto-electronic Functional Materials & Micro-nano Devices, Renmin University of China, Beijing 100872, China; Center for High Pressure Science and Technology Advanced Research, Shanghai 201203, China; Center for High Pressure Science and Technology Advanced Research, Shanghai 201203, China; Department of Physics and Beijing Key Laboratory of Opto-electronic Functional Materials & Micro-nano Devices, Renmin University of China, Beijing 100872, China; Department of Physics and Beijing Key Laboratory of Opto-electronic Functional Materials & Micro-nano Devices, Renmin University of China, Beijing 100872, China; Department of Physics and Beijing Key Laboratory of Opto-electronic Functional Materials & Micro-nano Devices, Renmin University of China, Beijing 100872, China; School of Physical Science and Technology, ShanghaiTech University, Shanghai 201210, China; ShanghaiTech Laboratory for Topological Physics, ShanghaiTech University, Shanghai 201210, China; Shanghai Key Laboratory of High-resolution Electron Microscopy, ShanghaiTech University, Shanghai 201210, China

**Keywords:** superconductivity, high pressure, borides

## Abstract

Since the discovery of superconductivity in MgB_2_ (*T*_c_ ∼ 39 K), the search for superconductivity in related materials with similar structures or ingredients has never stopped. Although about 100 binary borides have been explored, only a few of them show superconductivity with relatively low *T*_c_. In this work, we report the discovery of superconductivity up to 32 K, which is the highest *T*_c_ in transition-metal borides, in MoB_2_ under pressure. The *T*_c_ of MoB_2_ in the α phase can be well explained by theoretical calculations in the framework of electron-phonon coupling. Furthermore, the coupling between the *d* electrons of Mo and the out-of-plane Mo-phonon modes are the main driving force of the 32 K superconductivity of MoB_2_. Our study sheds light on the exploration of high-*T*_c_ superconductors in transition metal borides.

## Teaser

Discover the superconductivity up to 32 K in MoB_2_ under pressure and highlight roles of transitional metals in the high-Tc superconductors.

## INTRODUCTION

Superconductors with high transition temperature (high *T*_c_) are long-sought targets in the condensed matter physics and materials science communities. Materials with light elements [1–5] are especially favorable as they can provide high Debye frequency, which is proportional to the superconducting *T*_c_ according to Bardeen-Cooper-Schrieffer (BCS) theory [6]. In various light-element materials, metal borides have attracted much attention due to their unique crystal structure and rich physical phenomena. The discovery of superconductivity in MgB_2_ at 39 K in 2001 has reinforced the scientific importance of metal diborides (*M*B_2_) [1]. Nevertheless, after nearly two decades of exploration of superconductivity in the rich family of metal diborides, only a few *M*B_2_ show superconducting behavior. Furthermore, the *T*_c_ of these superconducting *M*B_2_ compounds are much lower than that of MgB_2_ (Table S1 within the online supplementary material).

**Figure 1.**
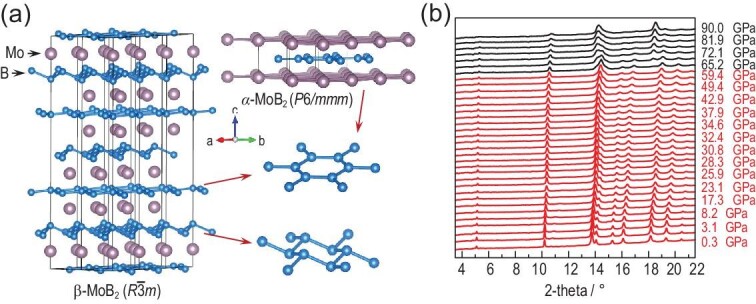
Crystal structure of MoB_2_ and its evolution with pressure. (a) Crystal structures of α-MoB_2_ (space group *P*6/*mmm*) and β-MoB_2_ (space group $R\bar{3}m$). (b) XRD patterns of MoB_2_ measured at room temperature with an increase of external pressure up to 90 GPa. The XRD wavelength λ is 0.6199 Å. Red and black curves are used to distinguish the structure transformation at 65.2 GPa.

Molybdenum diboride (MoB_2_) is unique among the *M*B_2_ family since it is the only material that has two structural forms: the α-MoB_2_ phase (AlB_2_ type, space group *P*6/*mmm*) [7–9] and β-MoB_2_ phase (CaSi_2_ type, space group $R\bar{3}m$) [10–12]. Although both phases of MoB_2_ have similar triangular Mo layers, their B atom arrangements are quite different. The B atoms in α-MoB_2_ constitute AA-staking two-dimensional (2D) graphitic boron layers. In contrast, there are two different kinds of B layers in β-MoB_2_ (Fig. 1(a)): one forms a nearly planar quasi-2D honeycomb lattice similar to those in α-MoB_2_ and MgB_2_; the other builds a buckled honeycomb network. As a result, the Mo atoms in β-MoB_2_ locate above the centers of hexatomic boron rings in the planar boron layers, but occupy the top sites right above B atoms in the buckled boron layers (Fig. 1(a)). MoB_2_ may thus provide a good material platform to make a comparative study with MgB_2_.

Pressure, as a conventional thermodynamic parameter, is a clean and powerful tool to tune the electronic properties of materials. It is possible to trigger structural and electronic transitions, subsequently inducing novel quantum phenomena [13]. Here we report on the structure and transport properties of MoB_2_ under various pressures. We find that MoB_2_ exhibits superconductivity under high pressure, reaching a *T*_c_ of 32 K around 100 GPa, which is the second highest *T*_c_ among all known boride superconductors. Synchrotron X-ray diffraction (XRD) measurements indicate that β-MoB_2_ transforms to α-MoB_2_ around 65 GPa. Although the compressed MoB_2_ has the same structure and comparable *T*_c_ with MgB_2_, the superconducting mechanism of the former is completely different from that of the latter. Theoretical calculation suggests that the *d* electrons and phonon modes of transition metal Mo atoms play utterly different roles in the emergence of superconductivity in contrast to the dominance of *p* electrons and phonon modes of B atoms in the superconductivity of MgB_2_.

## RESULTS AND DISCUSSION

We performed *in situ* XRD measurements on the structural evolution of MoB_2_ under various pressures. As shown in Fig. 1(b), all the diffraction peaks in the low-pressure range can be indexed well to a rhombohedral primitive cell of β-MoB_2_ (space group $R\bar{3}m$), and both the *a*-axis and *c*-axis lattice constants decrease with increasing pressure (Figs. S2 and S3 and Table S2 within the online supplementary material). The structure of β-MoB_2_ is robust until 65 GPa. Beyond ∼65 GPa, additional diffraction peaks emerge, indicating the occurrence of a structural phase transition. Meanwhile, we carried out a global minimization of the enthalpy of MoB_2_ under high pressure by combining *ab initio* total energy calculations and the Calypso technique on structure predictions [14]. At 90 GPa, we theoretically predict two structural forms (space groups *P*6/*mmm* and *I*4_1_/*amd*) of MoB_2_ with lower enthalpies than the $R\bar{3}m$ structure (Fig. S4a within the online supplementary material). No imaginary frequency is found in the phonon dispersions of these two predicted structures, suggesting their dynamical stability (Fig. S4c and S4d within the online supplementary material). We find that the XRD pattern at 90 GPa can be well refined by using the hexagonal α-MoB_2_ structure (space group *P*6/*mmm*; Figs S4b and S5 within the online supplementary material). These experimental and theoretical results suggest that, under high pressure, there is a structural phase transition from β-MoB_2_ to α-MoB_2_ with a critical pressure *P*_c_ ∼ 70 GPa. With the increasing pressure, the content of α-MoB_2_ increases and almost reaches 100% at 90 GPa (Fig. S2a within the online supplementary material).

**Figure 2.**
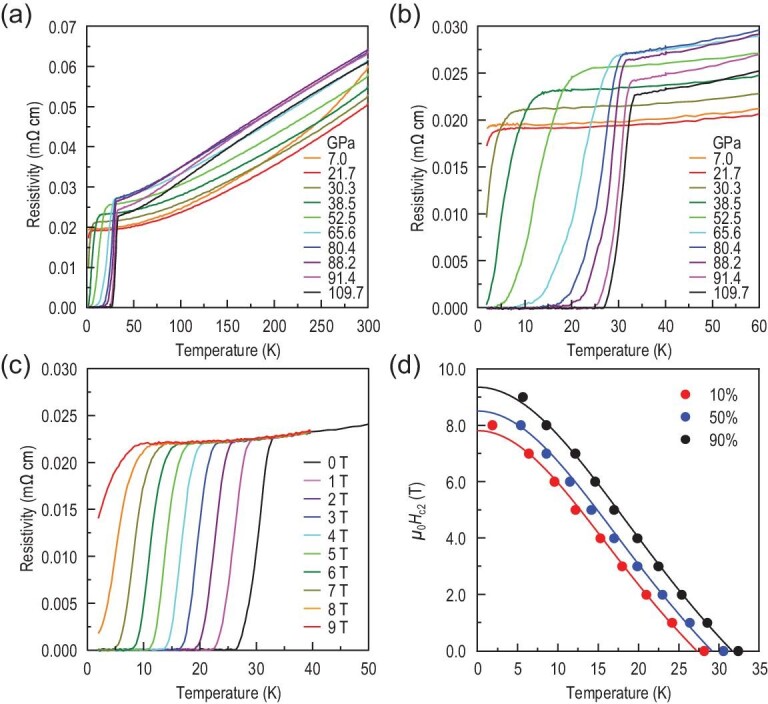
Transport properties of MoB_2_ as functions of pressure and magnetic fields in run IV. (a) Electrical resistivity ρ(*T*) of MoB_2_ as a function of temperature at different pressures. (b) Enlarged ρ(*T*) curves in the vicinity of the superconducting transition. (c) Electrical resistivity ρ(*T*) under various magnetic fields at 109.7 GPa. (d) Temperature dependence of the upper critical field μ_0_*H*_c2_(*T*) at 109.7 GPa. Here the *T*_c_ are determined at 10%, 50% and 90% of the normal state resistivity just above the onset superconducting transition temperature. The solid lines represent the fits using the Ginzburg-Landau formula.

Since MoB_2_ under high pressure possesses the same crystal structure as MgB_2_, a question arises naturally: is it possible to achieve superconductivity in MoB_2_ under high pressure? Hence, we measured the electrical resistivity ρ(*T*) of a β-MoB_2_ single crystal at various pressures. Figure 2(a) shows the typical ρ(*T*) curves for pressure up to 109.7 GPa. The ρ(*T*) curves display a metallic-like behavior in the whole pressure range. When the pressure increases to 21.7 GPa, a small drop of ρ is observed at the lowest measuring temperature (*T*_min_ = 1.8 K), as shown in Fig. 2(b). With further increasing pressure, zero resistivity is achieved at low temperature for *P* > 38.5 GPa, indicating the emergence of superconductivity. The superconducting *T*_c_ increases dramatically with pressure. Beyond the critical pressure (*P*_c_ = 70 GPa), where the structural phase transition happens, the growth of *T*_c_ slows down and the maximum *T*_c_ of 32.4 K is attained at *P* = 109.7 GPa, which is the highest pressure we can exert on the sample. At this pressure, the superconducting transition width Δ*T*_c_ [$= T\ (90\%\ \rho _{n}) - T\ (10\%\ \rho _{n})$] is about 4.2 K (Fig. 2(b)), which is rather sharp when compared with the large value of *T*_c_. Figure 2(c) demonstrates that at 109.7 GPa the resistivity drop is continuously suppressed with increasing magnetic field and it shifts to about 5 K at 9 T. Such behavior further confirms that the sharp decrease of ρ(*T*) should originate from a superconducting transition. The derived upper critical field μ_0_*H*_c2_(*T*) as a function of temperature *T* can be fitted well using the empirical Ginzburg-Landau formula (Fig. 2(d)) μ_0_*H*_c2_(*T*) = μ_0_*H*_c2_(0)(1 − *t*^2^)/(1 + *t*^2^), where *t* = *T*/*T*_c_ is the reduced temperature with zero-field superconducting *T*_c_. The fitted zero-temperature upper critical field μ_0_*H*_c2_(0) of MoB_2_ from the 90% ρ_*n*_ criterion can reach 9.4(1) T with *T*_c_ = 31.7(2) K, which is consistent with the measured value of 32.4 K.

**Figure 3.**
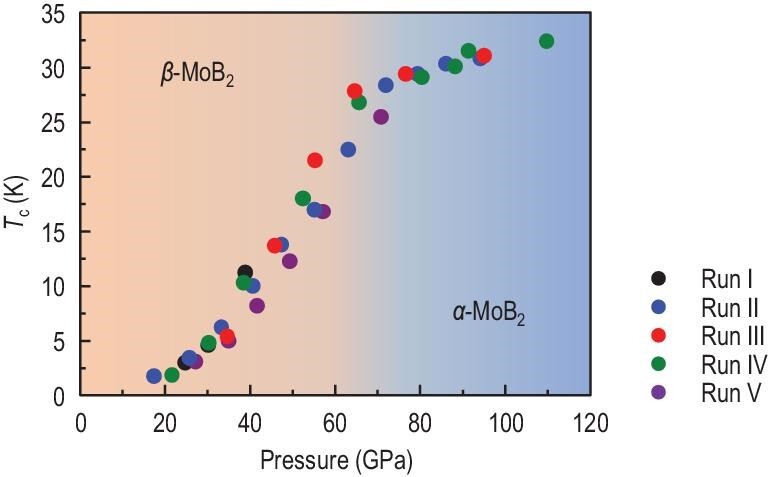
Phase diagram of MoB_2_. Pressure dependence of the superconducting transition temperature *T*_c_ for MoB_2_ up to 109.7 GPa in different runs. The values of *T*_c_ are determined from the high-pressure resistivity (90% ρ_*n*_ criterion).

The measurements on different samples of MoB_2_ for five independent runs provide consistent and reproducible results (Figs S6 and S7 within the online supplementary material), confirming this intrinsic superconductivity under pressure. The superconducting phase diagram of MoB_2_ as a function of the pressure is summarized in Fig. 3. It can be seen that the superconducting state emerges around 20 GPa, and then the *T*_c_ increases further with applied pressure. The *T*_c_ raises dramatically at a rate of 0.7 K/GPa in the range of 40 to 70 GPa, and beyond the structure-transition pressure (*P*_c_ ∼ 70 GPa) the growth of *T*_c_ slows down (0.1 K/GPa). The *T*_c_ of MoB_2_ rises to as high as ∼32 K at a pressure of 109.7 GPa and still does not exhibit the trend of saturation.

**Figure 4.**
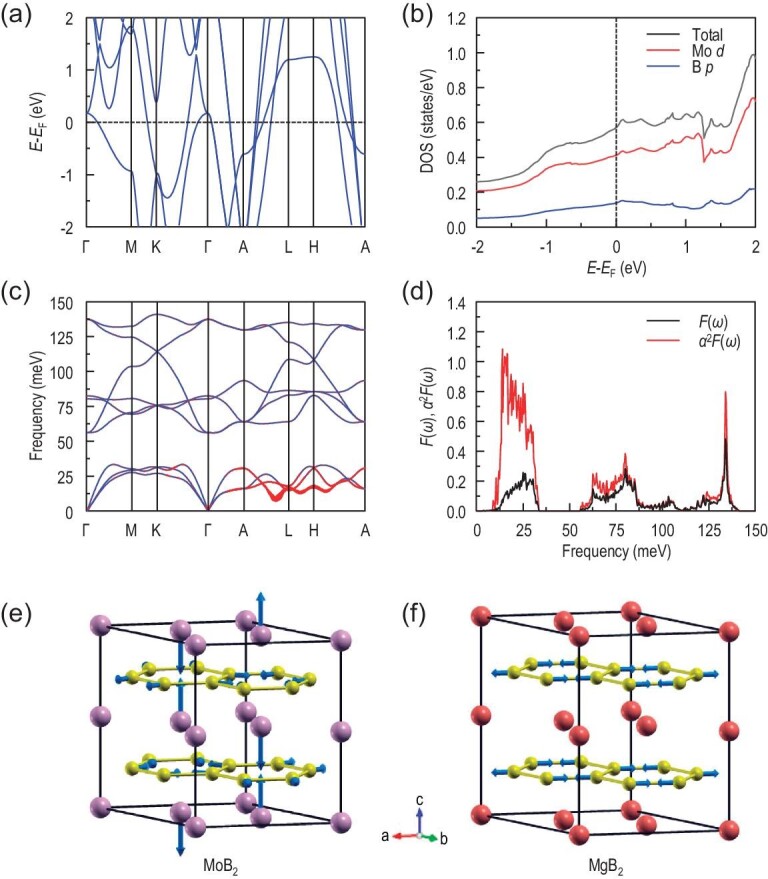
Calculated electronic structure and atomic displacements for the typical phonon modes of α-MoB_2_ under 90 GPa. (a) Electronic band structure. (b) Density of states *N*(*E*). (c) Phonon dispersion. The sizes of the red dots schematically denote the electron-phonon coupling strength λ_*qv*_. (d) Phonon density of states *F*(ω) (black line) and Eliashberg spectral function α^2^*F*(ω) (red line). (e) Atomic displacements of the lowest acoustic mode at the H point (Fig. 4(c)), which has a large EPC. A $\sqrt{3}\times \sqrt{3}\times 2$ supercell is employed. The arrows on Mo/B atoms indicate vibrational directions and magnitudes, where the arrows on the B atoms are magnified two times for clarity. (f) The in-plane B-B stretching mode for MgB_2_ at ambient pressure. The Mo, Mg and B atoms are represented by purple, red and yellow balls, respectively.

To explore the origin of the relatively high-*T*_c_ superconductivity in MoB_2_ under pressure, we performed density functional theory (DFT) [15,16] and density functional perturbation theory (DFPT) [17,18] calculations on MoB_2_ in the α phase at 90 GPa, for which the experimental *T*_c_ can reach ∼31.5 K at 91.4 GPa. The calculated band structure of α-MoB_2_ at 90 GPa along the high-symmetry paths of the Brillouin zone is shown in Fig. 4(a). There are several bands crossing the Fermi level (*E*_F_), indicating its metallic character. Based on the analysis of the total and partial density of states (Fig. 4(b)), Mo *d* orbitals (especially the *d*_*z*2_ orbital) have larger contributions than B *p* orbitals around *E*_F_. The phonon dispersion and the phonon density of states *F*(ω) for α-MoB_2_ at 90 GPa are displayed in Figs. 4(c) and 4(d), respectively. Because of the large mismatch between the atomic masses of Mo and B, there is a gap between the low-frequency acoustic branch contributed by Mo atoms and the high-frequency optical branch contributed by B atoms. Obviously, the acoustic modes along the A-L path and around the L and H points make great contributions to the electron-phonon coupling (EPC, represented by the red dots in Fig. 4(c)), which is also manifested in the Eliashberg spectral function α^2^*F*(ω) (red line in Fig. 4(d)). Figure 4(e) demonstrates a representative phonon mode of α-MoB_2_ at the H point at 90 GPa. For clarity, a $\sqrt{3}\times \sqrt{3}\times 2$ supercell is employed and the arrows on the B atoms are magnified two times. It can be seen that this phonon mode consists of the relative vibrations of Mo atoms perpendicular to the B-B honeycomb plane and the related in-plane breath-like vibrations of B atoms. The calculated total EPC constant λ is 1.60. Based on the McMillan-Allen-Dynes formula [19,20], we can then calculate the superconducting *T*_c_ with an effective screened Coulomb repulsion constant μ*. By adopting several values of μ* in a commonly used empirical range of 0.08 to 0.15 [19,20], we obtained *T*_c_ between 27.2 and 33.3 K (see Fig. S8 within the online supplementary material), showing good order of magnitude with the measured one (∼32 K). These theoretical calculations confirm that the observed superconductivity in MoB_2_ at high pressure should belong to the conventional BCS type.

Although α-MoB_2_ at high pressure shares the same crystal structure as MgB_2_ and owns a comparable superconducting *T*_c_ with that of MgB_2_ at ambient pressure [1], their electronic structures and superconducting ingredients are distinctly different. Firstly, for MgB_2_, the *p* orbitals of B atoms in the B-B honeycomb lattice contribute most around the Fermi level [21,22], while for α-MoB_2_ under high pressure, the states near *E*_F_ are dominated by the *d* electrons of Mo atoms (Fig. 4(b)) [23]. As a result, MoB_2_ has very large band dispersion along the *k*_z_ direction (such as the Γ-A path; Fig. 4(a)) and its Fermi surface shows a three-dimensional characteristic, i.e. without two-dimensional Fermi surfaces as in MgB_2_ [22,24] (Fig. S9 within the online supplementary material). Secondly, the main contribution to the EPC of α-MoB_2_ under high pressure derives from the low-frequency phonon branch (Fig. 4(c) and 4(d)); instead, the high-frequency branch plays a major role in MgB_2_ [25–28]. To be specific, for high-pressure α-MoB_2_, the out-of-plane phonon mode of Mo atoms couples strongly with Mo *d* electrons near *E*_F_ (Fig. 4(b) and 4(e)). In comparison, it is the in-plane B-B stretching mode in MgB_2_ that interacts intensively with the σ bond in the boron honeycomb lattice around *E*_F_ (Fig. 4(f)) [21]. Last but not least, previous theoretical studies suggest that the anisotropy in the EPC and the anharmonicity in the stretching phonon mode are very crucial for the high *T*_c_ of MgB_2_ [27–29]. Here, for α-MoB_2_ under high pressure, our calculations seem to coincide with the observed *T*_c_ without invoking the above two factors. These results reveal that the superconducting mechanism in high-pressure α-MoB_2_ is distinct from that in MgB_2_, suggesting the possibility of exploring new phonon-mediated high-*T*_c_ superconductors in transition metal borides.

In addition to the α phase of MoB_2_, we have also calculated the superconducting *T*_c_ of β-MoB_2_ using the same method. Nevertheless, the calculated *T*_c_ of β-MoB_2_ is always lower than 5 K and shows a decreasing tendency with pressure (see Fig. S10 within the online supplementary material), which are inconsistent with our experimental observations (Fig. 3). The small calculated *T*_c_ of β-MoB_2_ is related to its low electronic density of states *N*(*E*_F_) at the Fermi level (Fig. S10), which originates from the strong bonding between the Mo atoms and the B atoms in buckled boron layers. In the real β-MoB_2_ sample, the Mo atoms around the stacking fault or grain boundary can easily slide under pressure, which may drive some buckled boron layers to flat ones. As a result, the *N*(*E*_F_) could increase along with more planar boron layers (Fig. S10), hence facilitating the enhanced superconductivity. On the other hand, the pressure evolution of the strength of EPC in the β-MoB_2_ sample can be extracted from the fitting of the Raman spectrum by the Fano function [30–32], as shown in Fig. S11 within the online supplementary material. The Raman response to EPC manifests an asymmetric profile of the spectral line shape of Raman-active phonons. Explicitly, the inverse of the Fano asymmetric parameter |1/*q*| is proportional to the joint density of states, hence counting the strength of EPC. The representative fitting at 13.0 GPa is plotted in the inset of Fig. S11d. Combined with our first-principles calculations, we find that there are two Raman-active phonon modes that can provide strong EPC: one is the double-degenerated *E*_g_ mode around 200 cm^−1^ and the other is a *A*_g_ mode around 300 cm^−1^. Similar to the case in α-MoB_2_, both these two strong EPC modes come from the relative vibrations of Mo atoms, as shown in Fig. S11c. From the pressure dependent |1/*q*|, we note that the one from the *E*_g_ mode is nearly independent of pressure. However, the other one extracted from the *A*_g_ mode increases dramatically with increasing pressure, which presents compelling evidence of the enhanced EPC in MoB_2_ induced by increasing pressure. In addition, the *A*_g_ mode involves the vertical vibrations of Mo atoms relative to the B-B plane, which is similar to the corresponding phonon mode in α-MoB_2_ at high pressure (Fig. 4(e)), suggesting that more Mo atoms get rid of bonding with the B atoms in buckled layers with increasing pressure. The increased frequency and the enhanced EPC of this *A*_g_ mode as well as the boosted *N*(*E*_F_) mentioned above probably have a close connection with the increase of *T*_c_ in β-MoB_2_ in the phonon-mediated superconductivity regime.

## CONCLUSION

In summary, we found superconductivity up to 32 K in MoB_2_ under pressure. Although compressed α-MoB_2_ and MgB_2_ both possess the AlB_2_-type structures and comparable *T*_c_, the features of their electron-phonon couplings that mediate the superconductivity are quite different. In both MgB_2_ and α-MoB_2_, the active phonon modes directly modulate the active electronic bands, causing the strong electron-phonon coupling [33]. However, it is the out-of-plane Mo phonon that directly modulates the *d* electrons of Mo in α-MoB_2_, in comparison with the stretching B-phonon mode modulating the *p* orbitals of B in MgB_2_. Thus, not only do the electrons in light-element atom networks need to be considered, but the relatively localized electrons of metal atoms may also be worthy of attention in the search for high-*T*_c_ superconductors.

## MATERIALS AND METHODS

High-quality single crystals of MoB_2_ were grown by Al flux (see the online supplementary material). *In situ* high-pressure XRD and resistivity measurements were performed in a diamond anvil cell. We employed the swarm-intelligence-based CALYPSO structure prediction method [14] to find the energetically stable structures of MoB_2_ under high pressure. The electronic structure, phonon spectrum and EPC of AlB_2_-type α-MoB_2_ at 90 GPa were studied based on DFT [15,16] and DFPT [17,18] calculations. The details of the experiment are given in the online supplementary material.

## Note added

After we submitted this paper, we learned that pressure-induced superconductivity was observed in WB_2_ by another group [34]. Most of the data in that paper are consistent with our results [35]. We also observed superconductivity in itinerant antiferromagnet CrB_2_ via the application of external pressure [36].

## Supplementary Material

nwad034_Supplemental_File
