# Supplementary material for: Pressure-induced superconductivity at 32 K in MoB2
Source: Natl Sci Rev. 2023 Feb 14;10(5):nwad034. doi: 10.1093/nsr/nwad034 (PMC10228782; doi:10.1093/nsr/nwad034)
Supplement: nwad034_Supplemental_File [file nwad034_supplemental_file.pdf]

Pressure-induced Superconductivity at 32 K in MoB_2

Cuiying Pei[1#], Jianfeng Zhang[2#], Qi Wang[1,2,3#], Yi Zhao[1], Lingling Gao[1], Chunsheng Gong[2], Shangjie Tian[2], Ruitao Luo[2], Mingtao Li[4], Wenge Yang[4], Zhong-Yi Lu[2], Hechang Lei[2*], Kai Liu[2*], and Yanpeng Qi[1,3,5*]

[1]School of Physical Science and Technology, ShanghaiTech University, Shanghai 201210, China

[2]Department of Physics and Beijing Key Laboratory of Opto-electronic Functional Materials & Micro-nano Devices, Renmin University of China, Beijing 100872, China

[3]ShanghaiTech Laboratory for Topological Physics, ShanghaiTech University, Shanghai 201210, China

[4]Center for High Pressure Science and Technology Advanced Research, Shanghai 201203, China

[5]Shanghai Key Laboratory of High-resolution Electron Microscopy, ShanghaiTech University, Shanghai 201210, China

[#] These authors contributed to this work equally.

[*] Correspondence should be addressed to Y.P.Q. (qiyp@shanghaitech.edu.cn) or K.L. (kliu@ruc.edu.cn) or H.C.L. (hlei@ruc.edu.cn)

Materials and Methods

Sample synthesis, structural and composition characterizations. Single crystals of MoB_2 were grown by the Al flux method. Mo (99.5 %), B (99.9 %) and Al (99.99 %) with a molar ratio of Mo : B : Al = 1 : 2.5 : 73.3 were loaded in an alumina crucible. The mixture was heated up at 1773 K for 10 h, then it was cooled down to 1173 K with the rate of 30 K/h. The single crystal growth process was performed under high-purity argon atmosphere in the furnace. Finally, the MoB_2 single crystals were obtained by means of sodium hydroxide solution to remove the Al flux.

Experimental details of high-pressure measurements. *In situ* high pressure XRD measurements were performed at the beamline 15U at Shanghai Synchrotron Radiation Facility (λ = 0.6199 Å). Symmetric diamond anvil cell (DAC) with anvil culet sizes of 200 μm as well as Re gaskets were used. Mineral oil was used as pressure transmitting medium (PTM) and pressure was determined by the ruby luminescence method [1]. CeO_2 was used to calibrate the sample-detector distance and the orientation parameters of the detector. The two-dimensional diffraction images were analyzed using the FIT2D program [2]. Rietveld refinements on crystal structures under high pressure were performed by General Structure Analysis System (GSAS) and graphical user interface EXPGUI package [3,4].

High pressure resistivity measurements were performed in a nonmagnetic diamond anvil cell. A cubic BN/epoxy mixture layer was inserted between BeCu gaskets and electrical leads. Electrical resistivity was measured using the dc current in van der Pauw technique in Physical Property Measurement System (Dynacool, Quantum Design, T_{min} = 1.8 K). Pressure was measured using the ruby scale by measuring the luminescence from small chips of ruby placed in contact with the sample[1].

An *in situ* high-pressure Raman spectroscopy investigation of MoB_2 was performed using a Raman spectrometer (Renishaw inVia, UK) with a laser excitation wavelength of 532 nm and low-wavenumber filter. A symmetric DAC with anvil culet sizes of 200 μm was used, with silicon oil as the PTM.

Theoretical calculations. We employed the swarm-intelligence-based CALYPSO

structure prediction method [5] to find the energetically stable structures of MoB_2 under high pressure. Six independent searching missions at 90 GPa, which were respectively limited to 1, 2, 3, 4, 6, and 8 chemical formulae per unit cell, were carried out. The underlying enthalpy calculations were performed with the Vienna Ab-initio Simulation Package (VASP) [6]. The generalized gradient approximation (GGA) of Perdew-Burke-Ernzerhof (PBE) type was adopted for the exchange-correlation functional [7]. A kinetic energy cutoff of 360 eV was used for the plane-wave basis. A Monkhorst-Pack $\boldsymbol{k}$-point mesh with a grid spacing of 0.1 Å^{-1} was adopted for the Brillouin zone (BZ) sampling. The Gaussian smearing method with a width of 0.05 eV was used for the Fermi surface broadening. We also checked the enthalpy differences between different structures of MoB_2 (Supplemental Information Fig. S3a) by using the Quantum ESPRESSO (QE) package [8]. The consistent results were obtained, which insures the reliability of our calculations.

The electronic structure, phonon spectrum, and electron-phonon coupling (EPC) of AlB_2-type α-MoB_2 at 90 GPa were studied based on the density functional theory (DFT) [9,10] and density functional perturbation theory (DFPT) [11,12] calculations as implemented in the Quantum ESPRESSO (QE) package [8]. The interactions between electrons and nuclei were described by the norm-conserving pseudopotentials [13]. The GGA-PBE exchange-correlation functional was adopted. The kinetic energy cutoff of the plane-wave basis was set to be 80 Ry. A 24×24×24 $\boldsymbol{k}$-point mesh was used for the sampling of Brillouin zone (BZ). The Gaussian smearing method with a width of 0.004 Ry was employed for the Fermi surface broadening. In the structural optimization, both lattice constants and internal atomic positions were fully relaxed until the forces on all atoms were smaller than 0.0002 Ry/Bohr.

The superconductivity of AlB_2-type α-MoB_2 at 90 GPa was studied based on the electron-phonon coupling (EPC) theory as implemented in the EPW package [14], which uses the maximally localized Wannier functions (MLWFs) [15] and interfaces with QE [8]. We took the 6×6×6 $\boldsymbol{k}$-mesh and 6×6×6 $\boldsymbol{q}$-mesh as the coarse grids, and interpolated to the 48×48×48 $\boldsymbol{k}$-mesh and 24×24×24 $\boldsymbol{q}$-mesh dense grids, respectively. The EPC constant λ can be calculated either by the summation of the EPC strength $\lambda_{\boldsymbol{q}\nu}$

in the full BZ for all phonon modes or by the integral of the Eliashberg spectral function [16] $\alpha^2F(\omega)$ as following,

$$\lambda = \sum_{\boldsymbol{q}v} \lambda_{\boldsymbol{q}v} = 2 \int \frac{\alpha^2F(\omega)}{\omega} d\omega. \tag{1}$$

The Eliashberg spectral function $\alpha^2F(\omega)$ is defined as [16]

$$\alpha^2F(\omega) = \frac{1}{2\pi N(\varepsilon_{\mathrm{F}})} \sum_{\boldsymbol{q}v} \delta(\omega - \omega_{\boldsymbol{q}v}) \frac{\gamma_{qv}}{\hbar\omega_{\boldsymbol{q}v}}, \tag{2}$$

where $N(\varepsilon_{\mathrm{F}})$ is the density of states at the Fermi level ε_{F}, $\omega_{\boldsymbol{q}v}$ is the frequency of the v-th phonon mode at the wave vector $\boldsymbol{q}$, and $\gamma_{\boldsymbol{q}v}$ is the phonon linewidth. The superconducting transition temperature T_{c} can be calculated by substituting the EPC constant λ into the McMillan-Allen-Dynes formula [17,18],

$$T_c = \frac{\omega_{log}}{1.2} \exp[\frac{-1.04(1+\lambda)}{\lambda(1-0.62\mu^*)-\mu^*}], \tag{3}$$

where ω_{log} is the logarithmic average frequency defined as [17,18],

$$\omega_{log} = \exp[\frac{2}{\lambda} \int \frac{d\omega}{\omega} \alpha^2F(\omega)\ln(\omega)], \tag{4}$$

and μ^* is the effective screened Coulomb repulsion constant setting to an empirical value [19,20] of 0.1 in our calculations.

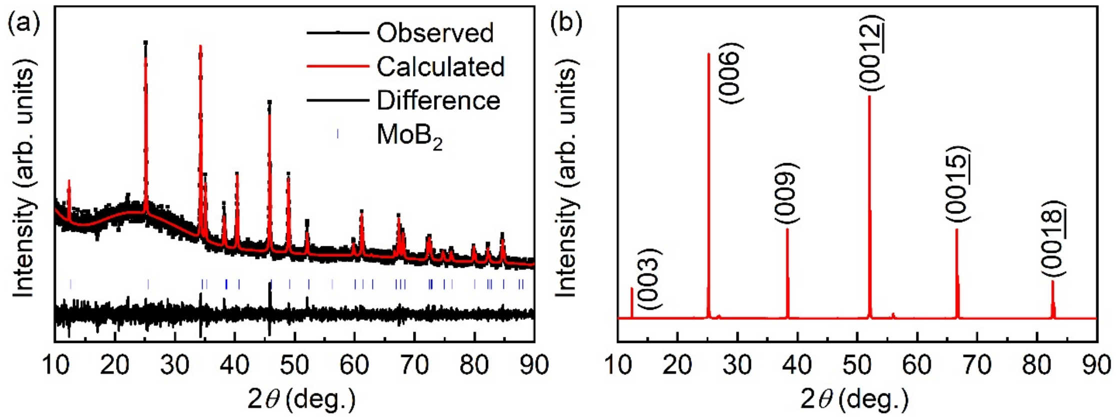

FIG. S1. Powder XRD pattern and Rietveld refinement of ground MoB_2 single crystals at ambient pressure. All peaks can be fitted well by using the structure of MoB_2 with the space group $R\bar{3}m$ (*No*. 166). (b) XRD pattern of MoB_2 single crystal at ambient pressure.

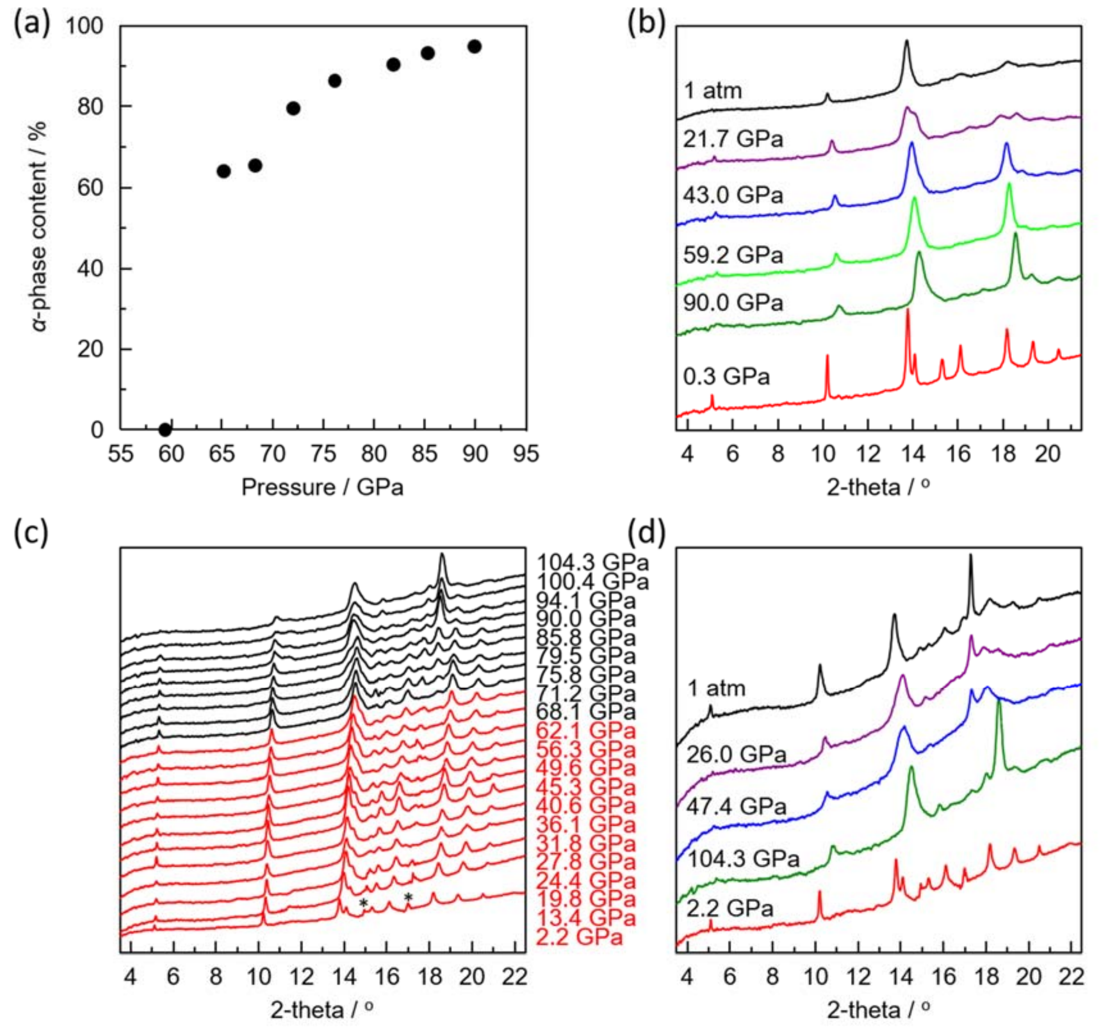

FIG. S2. Structure evolution of MoB_2 at different runs. (a) The α-phase content of MoB_2 as functions of pressure based on XRD results. (b) XRD patterns of MoB_2 under various

pressures at room temperature from 90.0 GPa decompressed to 1 atm and compared with that at original pressure of 0.3 GPa. (c) XRD patterns of MoB_2 under pressure up to 104.3 GPa at room temperature in run II. The red and black patterns distinguish phase transition from β-MoB_2 to α-MoB_2 with pressure over 62.1 GPa. The star symbol denotes reflection from impurity. (d) XRD patterns of MoB_2 under various pressures at room temperature from 104.3 GPa decompressed to 1 atm and compared with that at original pressure of 2.2 GPa.

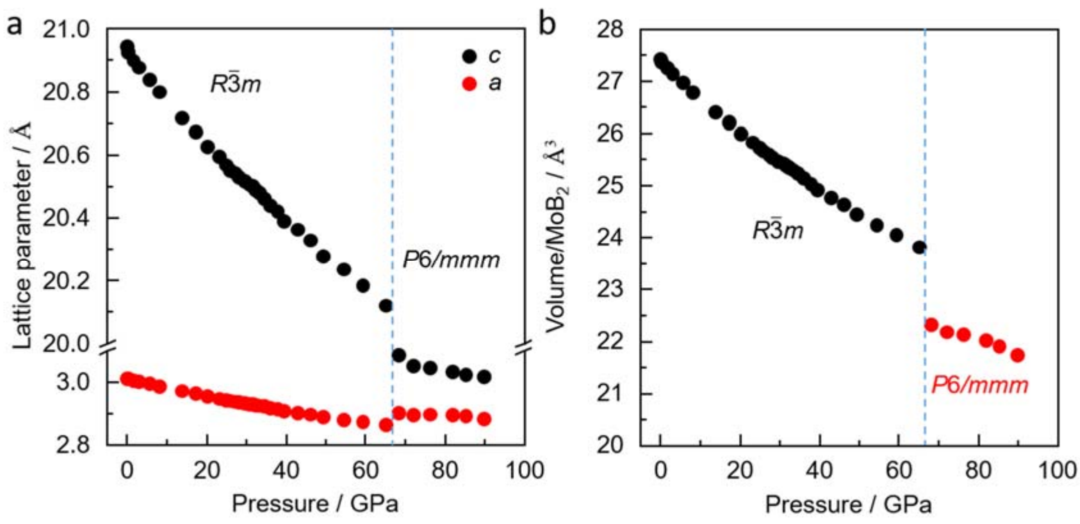

FIG. S3. Evolution of lattice parameters and volumes with pressure. Pressure dependence of lattice parameter *c*, *a* and lattice volume of MoB_2 relative to the $R\bar{3}m$ phase and *P*6/*mmm* phase.

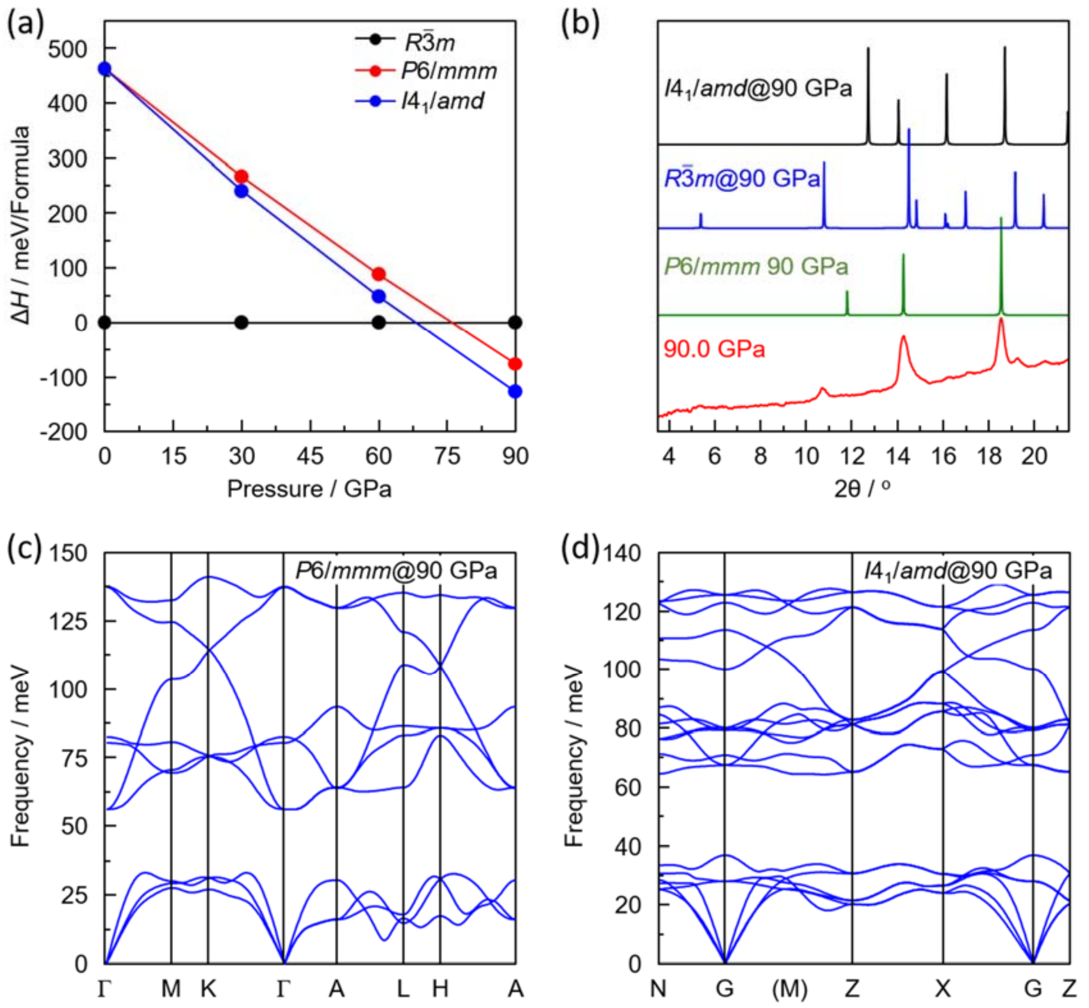

FIG. S4. Structural phase transition. (a) Calculated relative enthalpies of the *P6/mmm* and $I4_1/amd$ structures with respect to that of the $R\bar{3}m$ structure for MoB_2. Here, we selected the β-MoB_2 phase as the comparing standard (zero point): $\Delta H = H$ (specific phase) − H (β phase). (b) Typical simulated XRD patterns of MoB_2 with different structures in comparison with the measured spectrum at 90 GPa (red line). (c) Calculated phonon dispersion of the *P6/mmm*-structure MoB_2 at 90 GPa. (d) Calculated phonon dispersion of the $I4_1/amd$-structure MoB_2 at 90 GPa.

Both the *P6/mmm* and $I4_1/amd$ structures have lower enthalpies than the $R\bar{3}m$ structure (β-phase) for MoB_2 under high pressure. It is found that the XRD pattern at 90 GPa can be well refined by using the *P6/mmm* structural form of MoB_2 (α-phase). Observably, we could not refine with the $I4_1/amd$ structural form of MoB_2. Thus, there is a structural transition from β-MoB_2 to α-MoB_2 under high pressure.

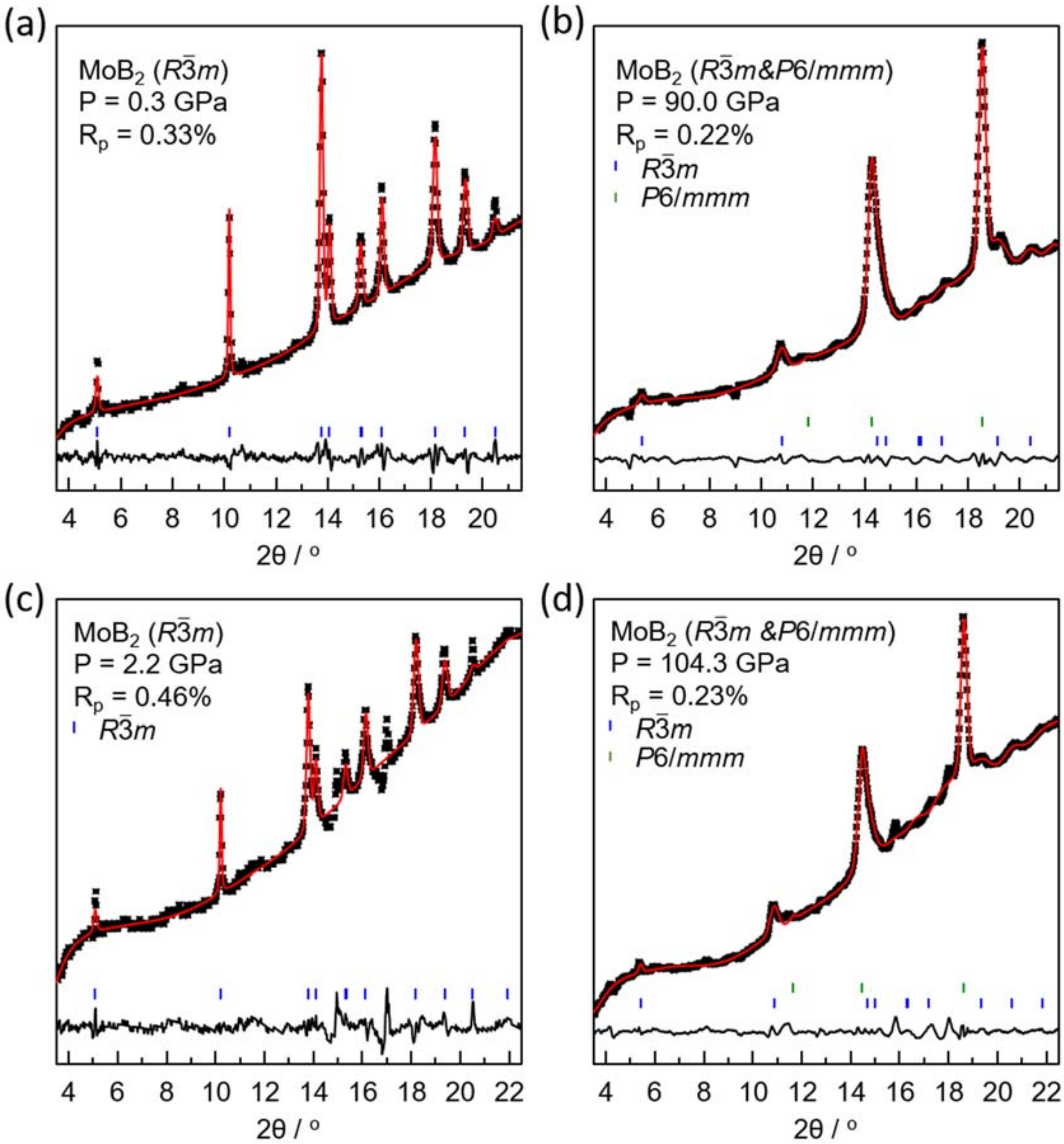

FIG. S5. Typical Rietveld refinement of MoB_2 at different runs. (a)-(b) 0.3 GPa and 90.0 GPa at run I. (c)-(d) 2.2 GPa and 104.3 GPa at run II. The experimental and calculated patterns are indicated by black stars and red lines, respectively. The solid lines shown at the bottom of the figures are the residual intensities. The vertical bars indicate peak positions of the Bragg reflections for MoB_2 in $R\bar{3}m$ (blue) and $P6/mmm$ (olive) space groups.

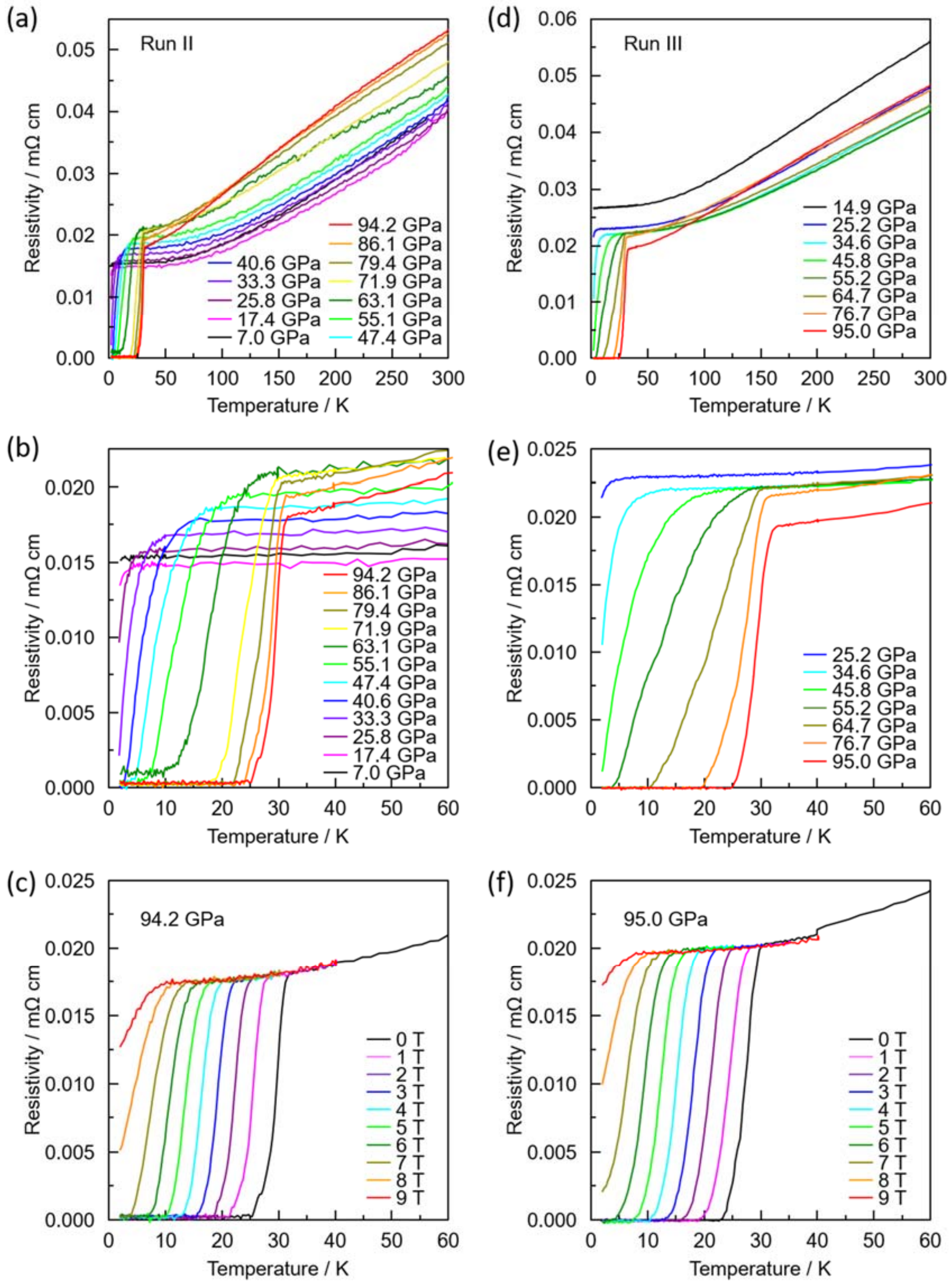

FIG. S6. Typical resistivity curves at different runs. (a) Electrical resistivity of MoB_2 as a function of temperature at different pressures in run II. (b) Temperature-dependent resistivity of MoB_2 in the vicinity of the superconducting transition in run II. (c) Temperature dependence of resistivity under different magnetic fields for MoB_2 at 94.2 GPa in run II. (d) Electrical resistivity of MoB_2 as a function of temperature at various pressures in run III. (e) Enlarged part of resistivity of MoB_2 in the vicinity of the

superconducting transition in run III. (f) Temperature dependence of resistivity under different magnetic fields for MoB_2 at 95.0 GPa in run III.

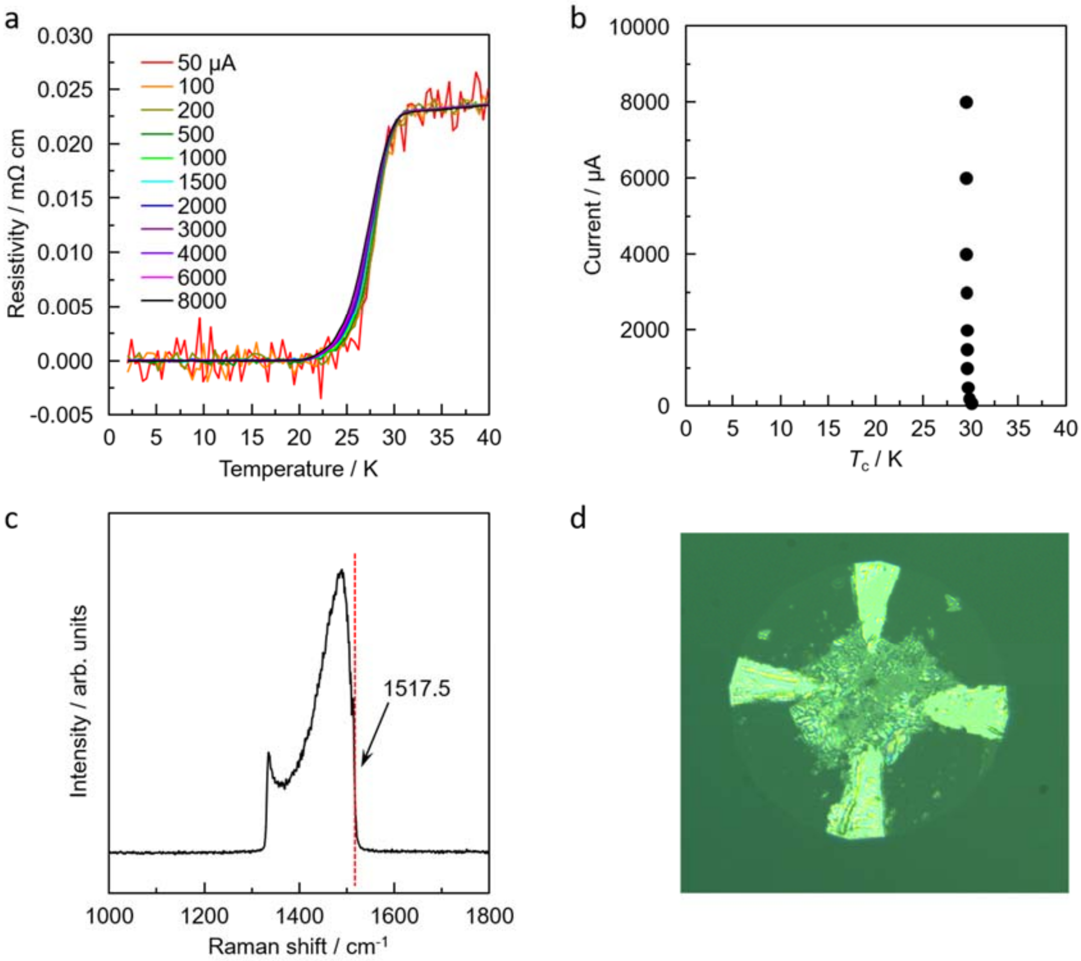

FIG. S7. (a) $\rho(T)$ under various excitation current at 89.4 GPa in the vicinity of the superconducting transition. (b) Temperature dependence of excitation current at 89.4 GPa. Here, the T_cs are determined at the 90% of the normal state resistivity just above the onset superconducting transition temperature, respectively. (c) The diamond Raman signal from which the pressure was determined. (d) An optical image of the sample loaded in the DAC.

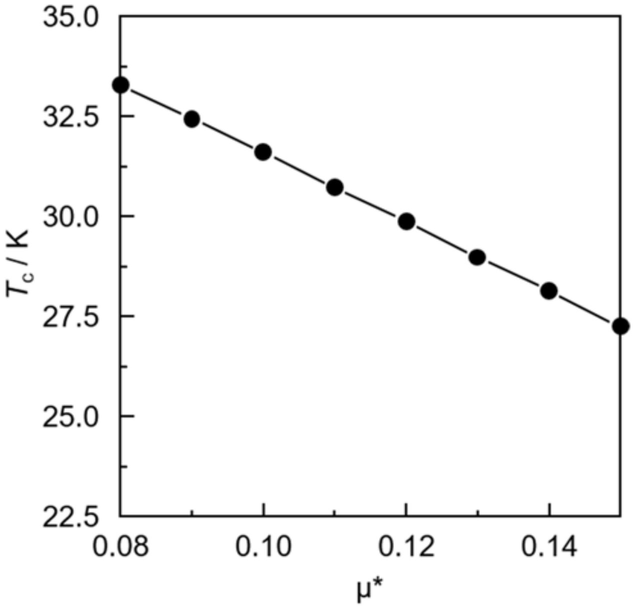

FIG. S8. Calculated superconducting T_c of α-MoB_2 at 90 GPa with different Coulomb repulsion constants μ * in a commonly-used empirical range of [0.08, 0.15].

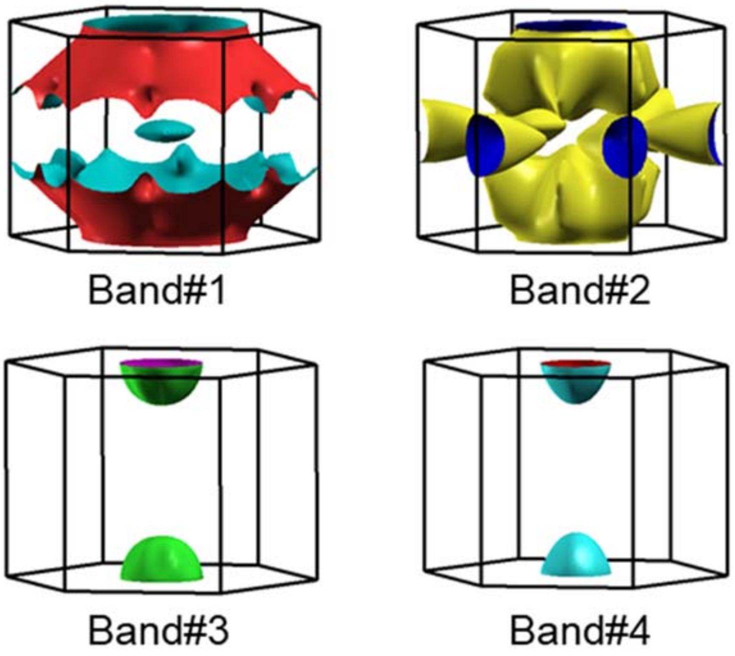

FIG. S9. Calculated Fermi surfaces of α-MoB_2 at 90 GPa. The pockets #1 and #2 mainly originate from Mo *d* orbitals, while the pockets of #3 and #4 primarily stem from B *p* orbitals. The Fermi surfaces of α-MoB_2 show three-dimensional characteristics and there is no such two-dimensional Fermi surface as in MgB_2.

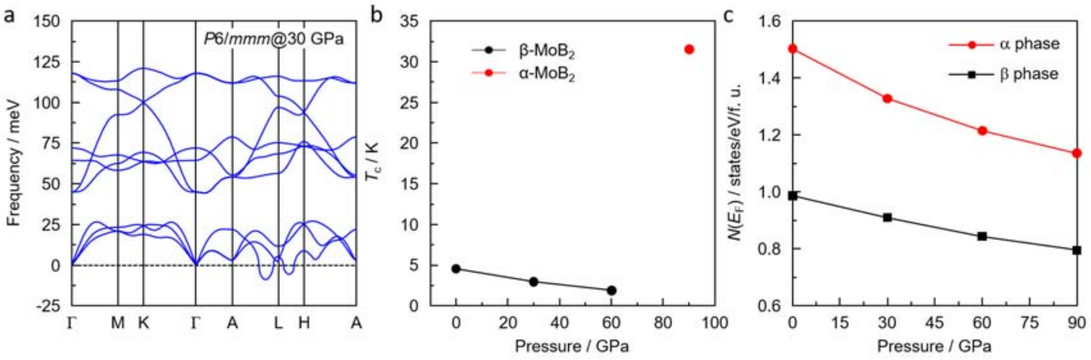

FIG. S10. (a) Phonon dispersion of α-phase MoB_2 at 30 GPa. Imaginary frequency is found in the phonon dispersions of α-phase MoB_2 at 30 GPa, suggesting the dynamical instability. So, the superconductivity below 70 GPa indeed come from β-phase MoB_2 (not α-phase MoB_2). (b) Calculated superconducting T_c of MoB_2 under different pressures. the calculated superconducting T_c of β-MoB_2 is always lower than 5 K and demonstrates a decrease tendency with pressure, which is inconsistent with our experimental observations. (c) Pressure dependence of calculated electronic density of states at the Fermi level $N(E_F)$ for MoB_2 in different structural phases.

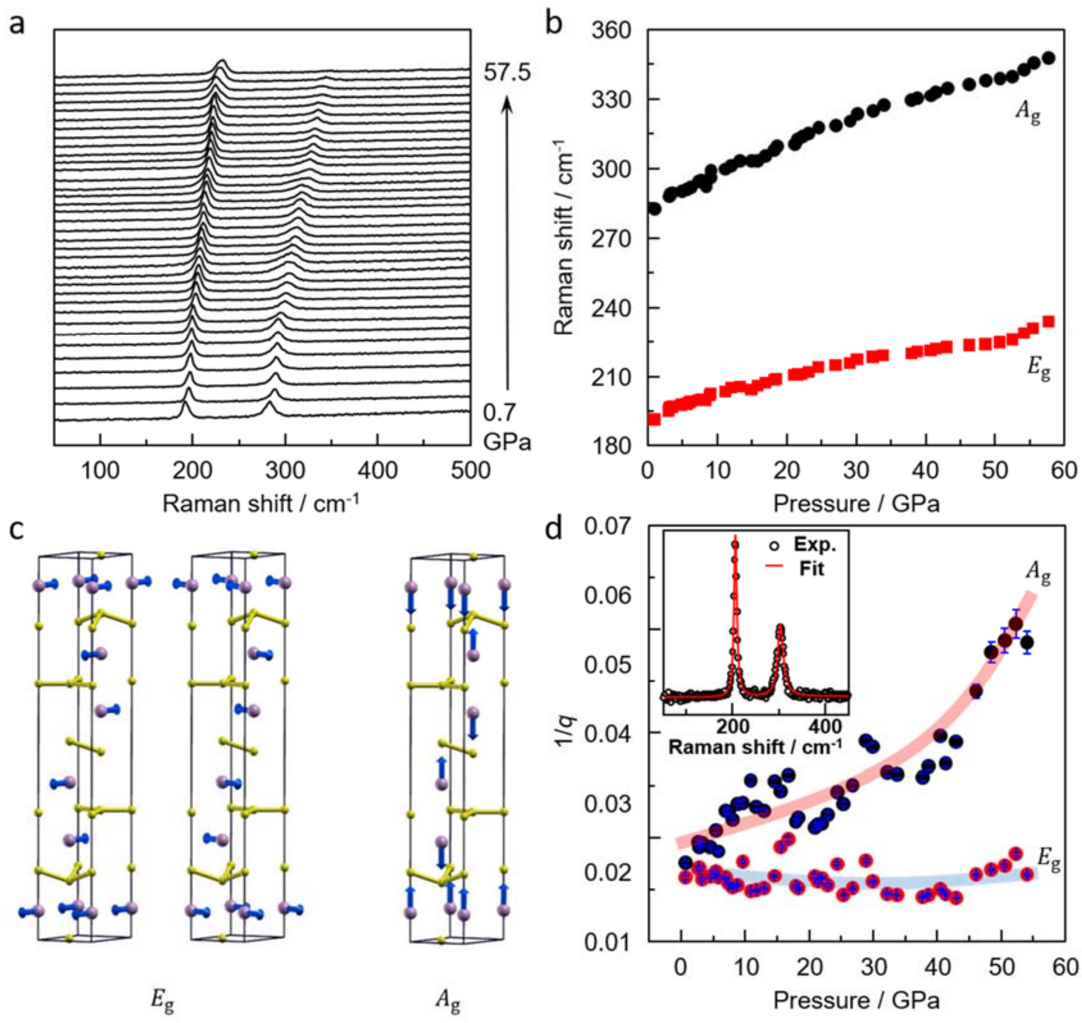

FIG. S11. (a) Raman spectra of β-phase MoB_2 under various pressures at room temperature. (b) Pressure-induced evolution of Raman-active modes. (c) Atomic displacement patterns of these two modes (E_g and A_g) from DFT calculations. (d) Pressure dependence of the inverse of Fano asymmetric parameter $1/q$ for two Raman-active phonon modes of β-MoB_2 in the pressure range of 0.7 to 60.0 GPa. Insert represents the Fano fitting curves for these two modes of MoB_2 at 13.0 GPa.

Table S1. List of superconducting borides.

Number	Compound	T_c / K	P / GPa	Structure	Ref.
1	MgB_2	39	1 atm	AlB_2	[21]
2	MgB_2	15	32	AlB_2	[22,23]
**3**	**MoB_2**	**27.8**	**64.7**	**$CaSi_2$**	**This work**
**4**	**MoB_2**	**32.4**	**109.7**	**AlB_2**	**This work**
5	$BeB_{2.75}$	0.7	1 atm	Not AlB_2	[24]
6	ZrB_2	5.5	1 atm	AlB_2	[25,26]
7	AgB_2	6.7(thin film)	1 atm	AlB_2	[27]
8	Ta_2B	3.12	1 atm	$CuAl_2$	[28]
9	Mo_2B	5.07	1 atm	θ-$CuAl_2$	[29]
10	Re_2B	2.8	1 atm		[28]
11	W_2B	3.22	1 atm	θ-$CuAl_2$	[30]
12	Re_3B	4.7	1 atm	Re_3B	[31]
13	Ru_7B_3	2.58	1 atm	Th_7Fe_3	[28]
14	ZrB	3.4	1 atm	NaCl	[28]
15	NbB	8.25	1 atm	α-TlI	[28]
16	MoB	0.5	1 atm	CrB	[28]
17	HfB	3.1	1 atm	NaCl	[28]
18	TaB	4.0	1 atm	α-TlI	[28,32]
19	YB_6	7.1	1 atm	CaB_6	[33]
20	LaB_6	5.7	1 atm	CaB_6	[33]
21	ThB_6	0.74	1 atm	CaB_6	[33]
22	NbB_6	3.0	1 atm		[28]
23	ScB_{12}	0.39	1 atm	UB_{12}	[33]
24	YB_{12}	4.7	1 atm	UB_{12}	[33]
25	ZrB_{12}	5.82	1 atm	UB_{12}	[33]
26	LuB_{12}	0.48	1 atm	UB_{12}	[33]
27	$YRuB_2$	7.8	1 atm	$LuRuB_2$	[34,35]
28	$LuRuB_2$	9.99	1 atm	$LuRuB_2$	[34,35]
29	$ScOsB_2$	1.34	1 atm	$LuRuB_2$	[34,35]
30	$YOsB_2$	2.22	1 atm	$LuRuB_2$	[34,35]
31	$LuOsB_2$	2.66	1 atm	$LuRuB_2$	[34,35]
32	Mo_2BC	7.5	1 atm	Mo_2BC	[36]
33	$Nb_2BN_{0.98}$	2.5	1 atm	Mo_2BC	[28]
34	YB_2C_2	3.6	1 atm	YB_2C_2	[37]
35	LuB_2C_2	2.4	1 atm	YB_2C_2	[37]
36	$Ca_{0.67}Pt_3B_2$	1.57	1 atm	$Ba_{0.67}Pt_3B_2$	[38]
37	$Sr_{0.67}Pt_3B_2$	2.78	1 atm	$Ba_{0.67}Pt_3B_2$	[38]
38	$Ba_{0.67}Pt_3B_2$	5.6	1 atm	$Ba_{0.67}Pt_3B_2$	[38]
39	$LaRh_3B_2$	2.82	1 atm	$CeCo_3B_2$	[39]
40	$LaIr_3B_2$	1.65	1 atm	$CeCo_3B_2$	[39]
41	$LuOs_3B_2$	4.67	1 atm	$CeCo_3B_2$	[39]
42	$ThRu_3B_2$	1.79	1 atm	$CeCo_3B_2$	[39]
43	$ThIr_3B_2$	2.09	1 atm	$CeCo_3B_2$	[39]
44	YRh_4B_4	11.34	1 atm	$CeCo_4B_4$	[40]
45	$NdRh_4B_4$	5.36	1 atm	$CeCo_4B_4$	[40]
46	$SmRh_4B_4$	2.51	1 atm	$CeCo_4B_4$	[40]

47	$ErRh_4B_4$	8.55	1 atm	$CeCo_4B_4$	[40]
48	$TmRh_4B_4$	9.86	1 atm	$CeCo_4B_4$	[40]
49	$LuRh_4B_4$	11.76	1 atm	$CeCo_4B_4$	[40]
50	$ThRh_4B_4$	4.34	1 atm	$CeCo_4B_4$	[40]
51	$DyRh_2Ir_2B_4$	4.64	1 atm	$CeCo_4B_4$	[41]
52	$HoRh_2Ir_2B_4$	6.41	1 atm	$CeCo_4B_4$	[41]
53	$ErIr_4B_4$	2.34	1 atm	$CeCo_4B_4$	[41]
54	$TmIr_4B_4$	1.75	1 atm	$CeCo_4B_4$	[41]
55	$ScRu_4B_4$	7.23	1 atm	$LuRh_4B_4$	[42]
56	YRu_4B_4	1.4	1 atm	$LuRh_4B_4$	[43]
57	$LuRu_4B_4$	2.06	1 atm	$LuRh_4B_4$	[43]
58	YRh_4B_4	10	1 atm	$LuRh_4B_4$	[43]
59	$ErRh_4B_4$	7.8	1 atm	$LuRh_4B_4$	[44]
60	YRu_2B_2C	9.7	1 atm	$LuNi_2B_2C$	[45]
61	$DyNi_2B_2C$	6.2	1 atm	$LuNi_2B_2C$	[28]
62	$HoNi_2B_2C$	8.7	1 atm	$LuNi_2B_2C$	[28]
63	$ErNi_2B_2C$	10.5	1 atm	$LuNi_2B_2C$	[28]
64	$TmNi_2B_2C$	11	1 atm	$LuNi_2B_2C$	[28]
65	$LuNi_2B_2C$	16.1	1 atm	$LuNi_2B_2C$	[28]
66	YNi_2B_2C	15.6	1 atm	$LuNi_2B_2C$	[28]
67	$ScNi_2B_2C$	15.6	1 atm	$LuNi_2B_2C$	[28]
68	$ThNi_2B_2C$	8	1 atm	$LuNi_2B_2C$	[28]
69	YPd_2B_2C	23	1 atm	$LuNi_2B_2C$	[28]
70	YPd_2B_2C	14.5	1 atm	$LuNi_2B_2C$	[28]
71	YPt_2B_2C	10	1 atm	$LuNi_2B_2C$	[28]
72	$LaPt_2B_2C$	10	1 atm	$LuNi_2B_2C$	[28]
73	$ThPt_2B_2C$	6.5	1 atm	$LuNi_2B_2C$	[28]
74	$PrPt_2B_2C$	6	1 atm	$LuNi_2B_2C$	[28]

Table S2. Structural parameters of MoB_2 under different pressures at room temperature.

	0.3 GPa	90.0 GPa	
Phase	Phase *β*	Phase *β*	Phase *α*
Crystal system	rhombohedral	rhombohedral	hexagonal
Space group	$R\bar{3}m$ (166)	$R\bar{3}m$ (166)	*P*6/*mmm* (191)
a	3.0102(1)	2.8591(2)	2.8841(3)
b	3.0102(1)	2.8591(1)	2.8841(3)
c	21.7340(3)	19.7962(4)	3.0173(2)
α	90	90	90
β	90	90	90
γ	120	120	120
atoms position	Wyckoff (*x y z*)	Wyckoff (*x y z*)	Wyckoff (*x y z*)
Mol	6*c* (0,0,0.0747)	6*c* (0,0,0.0753)	1*a* (0,0,0)
B1	6*c* (0,0,0.3353)	6*c* (0,0,0.3297)	2*d* (1/3,2/3,0.5)
B2	6*c* (0,0,0.1729)	6*c* (0,0,0.1944)	
Residuals[a] / %	R_{wp}: 0.46	R_{wp}: 0.28	
	R_p: 0.33	R_p: 0.22	

[a]Here $R_p = \Sigma||F_{obs}| - |F_{calc}||/\Sigma|F_{obs}|$ and $R_{wp} = (\Sigma[w(|F_{obs}|^2 - |F_{calc}|^2)^2]/\Sigma[w(|F_{obs}|^2)^2])^{1/2}$, where F_{obs} is the observed structure factor and F_{calc} is the calculated structure factor.

[1] H. K. Mao, J. Xu, and P. M. Bell, J. Geophys. Res. **91**, 4673 (1986).

[2] A. P. Hammersley, S. O. Svensson, M. Hanfland, A. N. Fitch, and D. Hausermann, High Press. Res. **14**, 235 (1996).

[3] A. C. Larson and R. B. V. Dreele, Los Alamos National Laboratory Report LAUR 86 (2004).

[4] B. Toby, J. Appl. Crystallogr. **34**, 210 (2001).

[5] Y. Wang, J. Lv, L. Zhu, and Y. Ma, Comput. Phys. Commun. **183**, 2063 (2012).

[6] G. Kresse and J. Furthmüller, Phys. Rev. B **54**, 11169 (1996).

[7] J. P. Perdew, K. Burke, and M. Ernzerhof, Phys. Rev. Lett. **77**, 3865 (1996).

[8] P. Giannozzi *et al.*, J. Phys. Condens. Matter **21**, 395502 (2009).

[9] P. Hohenberg and W. Kohn, Phys. Rev. **136**, B864 (1964).

[10] W. Kohn and L. J. Sham, Phys. Rev. **140**, A1133 (1965).

[11] F. Giustino, Rev. Mod. Phys. **89**, 015003 (2017).

[12] S. Baroni, S. de Gironcoli, A. Dal Corso, and P. Giannozzi, Rev. Mod. Phys. **73**, 515 (2001).

[13] N. Troullier and J. L. Martins, Phys. Rev. B **43**, 1993 (1991).

[14] J. Noffsinger, F. Giustino, B. D. Malone, C.-H. Park, S. G. Louie, and M. L. Cohen, Comput. Phys. Commun. **181**, 2140 (2010).

[15] A. A. Mostofi, J. R. Yates, G. Pizzi, Y.-S. Lee, I. Souza, D. Vanderbilt, and N. Marzari, Comput. Phys. Commun. **185**, 2309 (2014).

[16] G. M. Eliashberg, Sov. Phys. JETP **11**, 696 (1960).

[17] P. B. Allen, Phys. Rev. B **6**, 2577 (1972).
[18] P. B. Allen and R. C. Dynes, Phys. Rev. B **12**, 905 (1975).
[19] C. F. Richardson and N. W. Ashcroft, Phys. Rev. Lett. **78**, 118 (1997).
[20] K.-H. Lee, K. J. Chang, and M. L. Cohen, Phys. Rev. B **52**, 1425 (1995).
[21] J. Nagamatsu, N. Nakagawa, T. Muranaka, Y. Zenitani, and J. Akimitsu, Nature **410**, 63 (2001).
[22] S. Deemyad, T. Tomita, J. J. Hamlin, B. R. Beckett, J. S. Schilling, D. G. Hinks, J. D. Jorgensen, S. Lee, and S. Tajima, arXiv:cond-mat/0209270v1 (2002).
[23] I. Loa, K. Kunc, K. Syassen, and P. Bouvier, Phys. Rev. B **66**, 134101 (2002).
[24] D. P. Young, R. G. Goodrich, P. W. Adams, J. Y. Chan, F. R. Fronczek, F. Drymiotis, and L. L. Henry, Phys. Rev. B **65**, 180518(R) (2002).
[25] V. A. Gasparov, N. S. Sidorov, I. I. Zver'kova, and M. P. Kulakov, JETP Lett. **73**, 532 (2001).
[26] A. L. Ivanovskiĭ, Phys. Solid State **45**, 1829 (2003).
[27] R. Tomita, H. Koga, T. Uchiyama, and I. Iguchi, J. Phys. Soc. Japan **73**, 2639 (2004).
[28] C. Buzea and T. Yamashita, Supercond. Sci. Technol. **14**, R115 (2001).
[29] H. E. E., D. H., and K. J. M., J. Less-Common Met. **27**, 281 (1972).
[30] L. Leyarovska and E. Leyarovski, J. Less-Common Met. **67**, 249 (1979).
[31] A. Kawano, Y. Mizuta, H. Takagiwa, T. Muranaka, and J. Akimitsu, J. Phys. Soc. Japan **72**, 1724 (2003).
[32] R. Kiessling, Acta Chem. Scand. **3**, 603 (1949).
[33] B. T. Matthias, T. H. Geballe, K. Andres, E. Corenzwit, G. W. Hull, and J. P. Maita, Science **159**, 530 (1968).
[34] H. C. Ku and R. N. Shelton, Mat. Res. Bull. **15**, 1441 (1980).
[35] R. N. Shelton and H. C. Ku, Mat. Res. Bull. **15**, 1445.
[36] P. Lejay, B. Chevalier, J. Etourneau, P. Hagenmuller, and P. Peshev, Synth. Met. **4**, 139 (1981).
[37] T. Sakai, G.-Y. Adachi, and J. Shiokawa, J. Less-Common Met. **84**, 107 (1982).
[38] R. N. Shelton, J. Less-Common Met. **62**, 191 (1978).
[39] H. C. Ku, G. P. Meisner, F. Acker, and D. C. Johnston, Solid State Commun. **35**, 91 (1980).
[40] B. T. Matthias, E. Corenzwit, J. M. Vandenberg, H. Barz, and -. 74, Proc. Natl. Acad. Sci. USA **74**, 1334 (1977).
[41] H. C. Ku, B. T. Matthias, and H. Barz, Solid State Commun. **32**, 937 (1979).
[42] H. C. Ku, D. C. Johnston, B. T. Matthias, H. Barz, G. Burri, and L. Rinderer, Mat. Res. Bull. **14**, 1591 (1979).
[43] D. C. Johnston, Solid State Commun. **24**, 699 (1977).
[44] Y. Watanabe, H. Iwasaki, H. Iasaki, and Y. Muto, Acta Crys. **C42**, 1469 (1986).
[45] Y. Y. Hsu, H. C. Chiang, and H. C. Ku, J. Appl. Phys. **83**, 6789 (1998).
